# Contrast-enhanced computed tomography-based radiomics nomogram for predicting HER2 status in urothelial bladder carcinoma

**DOI:** 10.3389/fonc.2024.1427122

**Published:** 2024-08-14

**Authors:** Jiao Peng, Zhen Tang, Tao Li, Xiaoyu Pan, Lijuan Feng, Liling Long

**Affiliations:** ^1^ Department of Radiology, The First Affiliated Hospital of Guangxi Medical University, Nanning, Guangxi, China; ^2^ Department of Radiology, Liuzhou Workers Hospital, The Fourth Affiliated Hospital of Guangxi Medical University, Liuzhou, Guangxi, China; ^3^ Department of Urology, Liuzhou Workers Hospital, The Fourth Affiliated Hospital of Guangxi Medical University, Liuzhou, Guangxi, China

**Keywords:** urothelial bladder carcinoma, HER2, contrast-enhanced CT, radiomics, nomogram

## Abstract

**Objective:**

To evaluate the performance of a clinical-radiomics model based on contrast-enhanced computed tomography (CE-CT) in assessing human epidermal growth factor receptor 2 (HER2) status in urothelial bladder carcinoma (UBC).

**Methods:**

From January 2022 to December 2023, 124 patients with UBC were classified into the training (n=100) and test (n=24) sets. CE-CT scans were performed on the patients. Univariate and multivariate analyses were conducted to identify independent predictors of HER2 status in patients with UBC. We employed eight machine learning algorithms to establish radiomic models. A clinical-radiomics model was developed by integrating radiomic signatures and clinical features. Receiver operating characteristic curves and decision curve analysis (DCA) were generated to evaluate and validate the predictive capabilities of the models.

**Results:**

Among the eight classifiers, the random forest radiomics model based on CE-CT demonstrated the highest efficacy in predicting HER2 status, with area under the curve (AUC) values of 0.880 (95% CI: 0.813–0.946) and 0.814 (95% CI: 0.642–0.986) in the training and test sets, respectively. In the training set, the clinical-radiomics model achieved an AUC of 0.935, an accuracy of 0.870, a sensitivity of 0.881, and a specificity of 0.854. In the test set, the clinical-radiomics model achieved an AUC of 0.857, an accuracy of 0.760, a sensitivity of 0.643, and a specificity of 0.900. DCA analysis indicated that the clinical-radiomics model provided good clinical benefit.

**Conclusion:**

The radiomics nomogram demonstrates good diagnostic performance in predicting HER2 expression in patients with UBC.

## 1 Introduction

Bladder cancer is the most prevalent type of tumor in the urinary system, commonly originating from the bladder mucosa (1). Urothelial bladder carcinoma (UBC), one of the most common types of bladder cancer globally, is classified into two categories based on the muscle invasion status: non-muscle-invasive bladder cancer (NMIBC), which is found in approximately 75% of patients, and muscle-invasive bladder cancer (MIBC), which represents approximately 25% of cases (2, 3). MIBC is often associated with a poor prognosis, characterized by a 5-year overall survival rate of below 50% (4). Although NMIBC has a better prognosis, it is associated with a high recurrence rate, and approximately 25%-30% of all NMIBC cases finally progress to MIBC (5).

Human epidermal growth factor receptor 2 (HER2/ERBB2) is a protein that plays a significant role in various cellular activities, including cell growth, proliferation, and differentiation (6, 7). It is found on the surface of certain cell types and belongs to the family of epidermal growth factor receptors. The expression of HER2 in UBC has significant clinical implications. Studies have shown that UBC patients with HER2 overexpression have a poorer prognosis than those with low HER2 expression (8, 9). Anti-HER2 therapies, such as disitamab vedotin (RC48), have shown significant clinical benefits and a manageable safety profile in locally advanced or metastatic HER2-positive patients with UBC (10). HER2 can independently predict prognosis in patients with NMIBC receiving bacillus Calmette-Guérin (BCG) therapy. Patients with HER2-positive, high-risk NMIBC have an elevated risk of disease recurrence and progression with BCG therapy. Therefore, aggressive targeted strategies, such as HER2-targeted therapies, should be considered for these patients (11). HER2 status is now considered an important biomarker in UBC for prognosis, treatment selection, and decision-making.

Currently, the expression of HER2 is examined by immunohistochemistry (IHC) or fluorescence *in situ* hybridization (FISH) in pathological tissue obtained by biopsy or surgical resection. Both IHC and FISH require invasive procedures to obtain specimens, which may lead to risks such as infection, bleeding, and other complications. Additionally, IHC and FISH have disadvantages such as expensive test kits, time-consuming procedures, and delays in treatment decision-making (12). Hence, there exists an immediate and crucial demand for a noninvasive and precise technique to preoperatively evaluate HER2 status in patients with UBC.

Radiomics combines medical imaging, computational analysis, and machine learning to unlock hidden information within medical images, ultimately aiming to improve patient care and outcomes through more precise diagnosis, prognosis, and treatment planning. In recent years, some studies have highlighted the potential of radiomics in predicting the expression of HER2 among various tumors (13, 14). Yu et al. suggested that MRI-based radiomics can better predict HER2 expression in patients with UBC and can be used for prognosis assessment and clinical decision-making (15). However, no prior investigations have focused on CT-based radiomics for assessing HER2 status in patients with UBC. Computed tomography (CT) is a common and crucial method for diagnosing, evaluating treatment, and conducting postoperative follow-ups for bladder cancer. It has become widely adopted in clinical practice. In recent years, radiomics based on CT images has developed rapidly in bladder cancer, demonstrating potential in guiding clinical decisions (2, 16, 17). Consequently, our research aims to evaluate the efficacy of CT-based radiomics in assessing HER2 expression in patients with UBC.

## 2 Materials and methods

### 2.1 Patients and data acquisition

**Figure 1:**
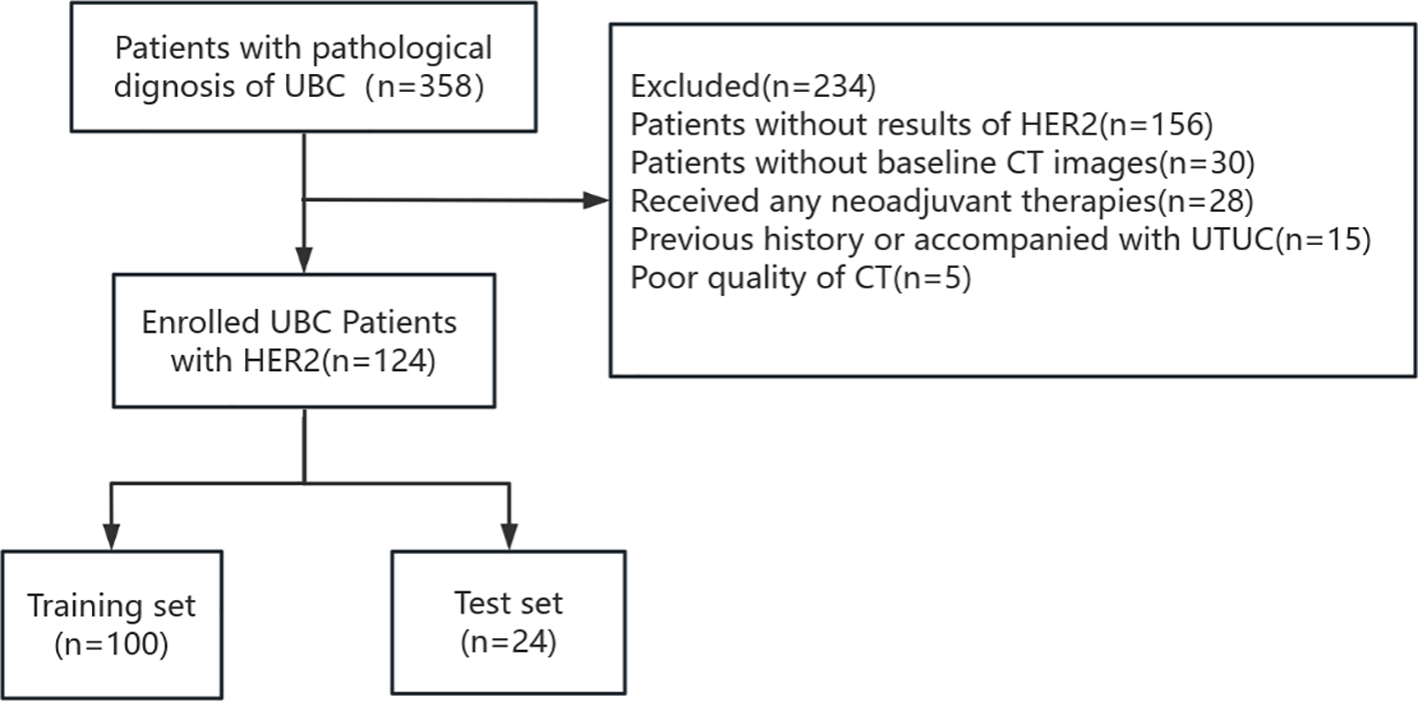
Flow chat of patient selection.

This retrospective study obtained approval from the Ethics Committee of the First Affiliated Hospital of Guangxi Medical University, and the requirement for informed consent from patients was waived. We included 124 patients with UBC in our cohort between January 2022 and December 2023. The patient selection flowchart is shown in **Figure 1**.

The inclusion criteria were: 1) Postoperative pathologically confirmed UBC at our institution; 2) HER2 expression in postoperative tissue specimens determined by IHC; and 3) available preoperative contrast-enhanced CT (CE-CT) images and complete clinical data.

The exclusion criteria were: 1) Preoperative CE-CT performed more than 30 days before surgery; 2) chemotherapy, radiotherapy, or targeted therapy were performed before CT scanning; and 3) previous history or concurrent upper tract urothelial carcinoma.

Clinical and imaging factors collected included age, gender, smoking history, clinical T-stage, tumor stalk, and tumor size. A tumor stalk is a structure that connects a tumor mass to the surrounding tissue or organ. Kang et al. reported that the tumor stalk appears as a hypoattenuating or hyperattenuating structure that contacts the bladder wall at the base of the tumor (18).

### 2.2 Evaluation of HER2 expression

Experienced pathologists evaluated all samples, selecting a morphologically representative section. HER2 protein expression in UBC was assessed using immunohistochemistry (IHC) with an anti-HER2/neu (4B5) rabbit monoclonal antibody (Ventana; Roche Diagnostics) on an automated IHC staining instrument (Ventana Benchmark XT, Roche). HER2 staining was classified using a 4-level system based on the 2018 American Society of Clinical Oncology/College of American Pathologists (ASCO/CAP) guidelines (19), as follows: 0: Negative membrane staining or <10% of cancer cells with incomplete/weak membrane staining; 1+: ≥10% of cancer cells with incomplete and weak membrane staining; 2+: ≥10% of cancer cells with weak to moderate complete membrane staining or <10% of tumor cells with strong and complete membrane staining; and 3+: ≥10% of cancer cells with strong/complete membrane staining. HER2 status was categorized as HER2 positive (IHC2+/3+) and HER2 negative (IHC0, 1+).

### 2.3 CT acquisition

CT scans were performed using various scanners, including the 256-row CT (Revolution CT, GE, USA), 64-row CT (LightSpeed VCT 64, GE, USA), SOMATOM Force (Siemens, Germany), 64-row multidetector CT scanner (Somatom 64, Siemens AG, Germany), and dual-source MDCT scanner (Somatom Definition, Siemens). Parameters were tube voltage: 100–120 kV; tube current: 250–300 mA; matrix 512×512; pitch: 0.6–1.25 mm; and reconstruction slice thickness: 1 mm, 1.25 mm, or 2 mm. Patients were advised to fast for 6–8 hours before the examination and to drink water to ensure adequate bladder filling. The patient assumes a supine position, and the scanning range is from the upper pole of the kidney to the symphysis pubis, starting with a plain CT scan of the urinary system followed by contrast agent injection (iohexol, iodine content 350 mg/mL) using a high-pressure injector. Images were acquired at 30 seconds for the corticomedullary phase, 60 seconds for the nephrographic phase, and 5 minutes for the excretory phase.

### 2.4 Lesion segmentation and CT features extraction

**Figure 2:**
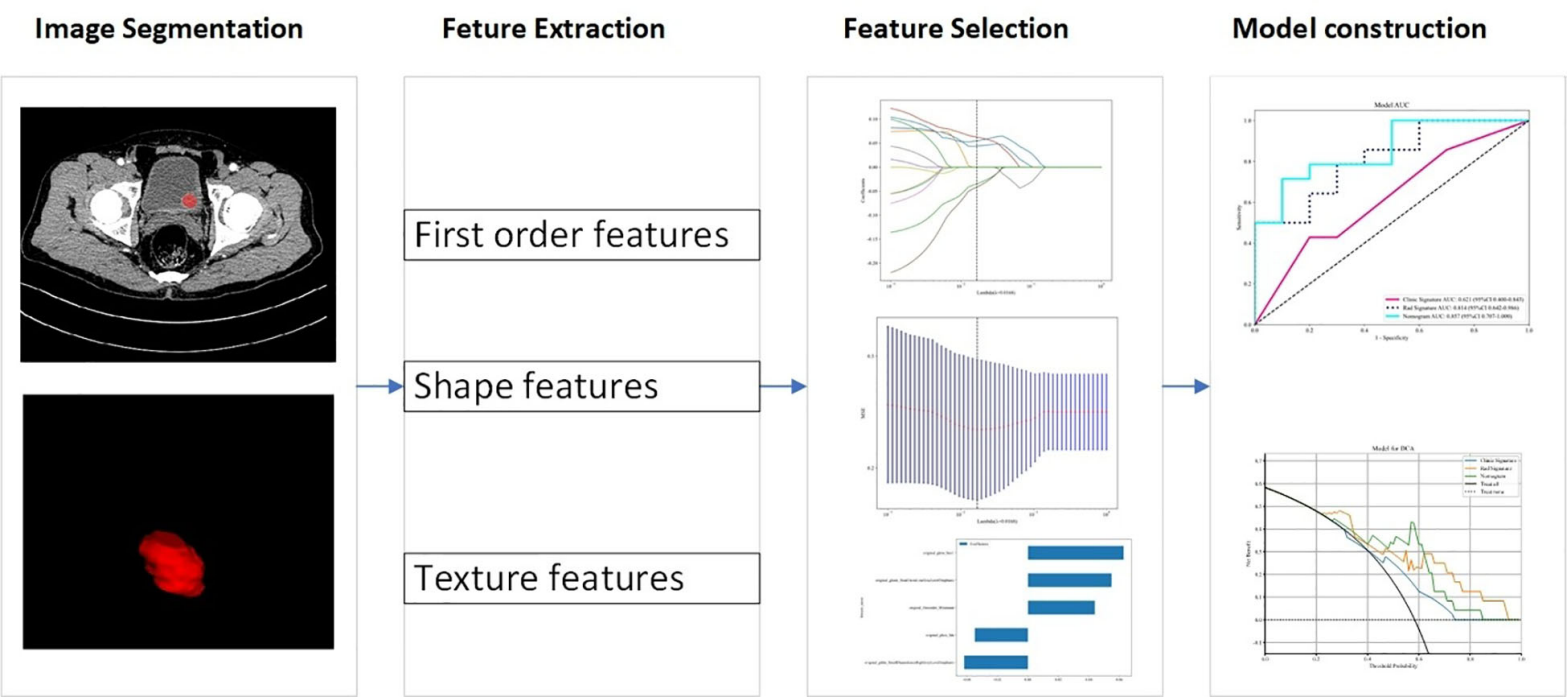
Workflow of radiomic analysis in this study.

**Figure 2** illustrates the workflow of the radiomics analysis. CE-CT images in the corticomedullary phase were analyzed. In our study, all CT features involved in extraction and selection were completed using the “One-key AI” platform. Before image segmentation, all CT images were resampled to a uniform image spacing of 1.0 mm in three anatomical directions due to different pixel sizes and slice thicknesses of various CT scanners. The region-of-interest of tumor lesions was segmented slice by slice using ITK SNAP software (www.itksnap.org). Two radiologists with 5 and 7 years of experience in genitourinary system diagnosis manually segmented the UBC lesions, blinded to HER2 status. An interclass correlation coefficient value ranging from 0.75 to 1 indicated good agreement.

A total of 1130 radiomics features were extracted from CT images, classified into three categories: geometric, intensity, and texture features. Geometric features assessed three-dimensional tumor morphology, intensity features described the distribution and variation of voxel intensities, and texture features captured various mathematical methods, including statistical measures, frequency domain analysis, and spatial domain analysis. Common texture features include gray-level run length matrix (GLRLM), gray-level co-occurrence matrix (GLCM), gray-level size zone matrix (GLSZM), and fractal features. Texture features refer to quantitative measures that capture the spatial arrangement and variation of pixel intensities within an image.

### 2.5 Feature selection and model building

We used the U test (significance threshold of p<0.01) for feature selection to recognize the most relevant features. For highly repeatable radiomic features, Spearman correlation analysis was conducted, removing features with a correlation coefficient exceeding 0.90, with one of them randomly removed. For signature construction, we utilized the least absolute shrinkage and selection operator (LASSO) regression model with 10-fold cross-validation. The final selected features were inputted into machine learning models, including support vector machine (SVM), random forest (RF), logistic regression (LR), multilayer perceptron (MLP), extremely randomized trees (ExtraTrees), light gradient boosting machine (LightGBM), extreme gradient boosting (XGBoost), and K-nearest neighbor (KNN). Five-fold cross-validation was conducted to construct the final Rad signature. The model with the highest area under the curve (AUC) in the test set was considered optimal.

Clinical factors were assessed using T-tests, Mann-Whitney U tests, or chi-square tests. Univariate and multivariate analyses explored independent predictors for HER2 status in patients with UBC. Clinical factors with p<0.05 were used to construct a clinical model. The radiomic nomogram was constructed by integrating the radiomics score with the clinical signature. Receiver operating characteristic (ROC) curves evaluated the diagnostic performance of the predictive models, and decision curve analysis (DCA) assessed their clinical usefulness.

### 2.6 Statistical analysis

Statistical analyses were performed using SPSS (IBM SPSS version 26.0) and the “One-key AI” platform (available at: https://www.medai.icu). Continuous variables were assessed using the Mann-Whitney U test or Student’s t-test, depending on their distribution. Categorical variables were analyzed using chi-square tests. Bilateral p<0.05 were considered statistically significant. ROC curves were plotted, and the AUC was computed to evaluate the diagnostic efficacy of the models. DCA was used to assess the clinical efficacy of the predictive models. The DeLong test was employed to compare the AUCs of the three models.

## 3 Results

### 3.1 Clinical characteristics

The basic clinical data of patients with UBC in our study are presented in **Tables 1**, **2**. We analyzed 124 patients diagnosed with UBC, categorizing them into two groups based on HER2 expression levels: HER2 positive (73 cases) and HER2 negative (51 cases). After random grouping, 100 patients were allocated to the training group, while the test group comprised 24 patients. Gender and stalk showed significant differences between the HER2-positive and HER2-negative groups on multivariable analysis (p<0.05).

**Table 1:** Baseline characteristics of patients in the training and test cohorts.

Characteristic	Training cohort (n=100)			Test cohort (n=24)		
	HER2-positive (n=59)	HER2-negative (n=41)	p	HER2-positive (n=14)	HER2-negative (n=10)	p
age	64.64 ± 11.56	64.24 ± 12.78	0.871	59.71 ± 13.60	64.50 ± 7.89	0.330
diameter	3.86 ± 1.68	3.90 ± 2.70	0.309	2.89 ± 1.44	3.68 ± 2.06	0.412
Clinical T stage			0.028			0.831
<T2	23(38.98)	26(63.41)		8(57.14)	7(70.00)	
≥T2	36(61.02)	15(36.59)		6(42.86)	3(30.00)	
stalk			0.001			0.339
NO	50(84.75)	22(53.66)		12(85.71)	6(60.00)	
YES	9(15.25)	19(46.34)		2(14.29)	4(40.00)	
gender			0.056			1.000
NO	6(10.17)	11(26.83)		2(14.29)	1(10.00)	
YES	53(89.83)	30(73.17)		12(85.71)	9(90.00)	
smoke			0.106			0.464
NO	28(47.46)	27(65.85)		6(42.86)	2(20.00)	
YES	31(52.54)	14(34.15)		8(57.14)	8(80.00)	

**Table 2:** Univariate and multifactor logistic regression analysis of diagnostic factors.

Characteristic	Univariable logistic regression		Multivariable logistic regression	
	OR (95% CI)	*p* value	OR (95% CI)	*p* value
age	1.001 (0.994–1.008)	0.871		
gender	1.331 (1.074–1.649)	0.029	1.328 (1.088–1.623)	0.021
diameter	0.998 (0.960–1.038)	0.935		
smoke	1.197 (1.017–1.409)	0.070		
Clinical T stage	1.267 (1.079–1.487)	0.016	1.173 (1.003–1.373)	0.094
stalk	0.689 (0.579–0.819)	0.001	0.726 (0.610–0.865)	0.003

OR, odds ratio; CI, confidence interval.

### 3.2 Feature selection and optimal machine learning algorithm

**Figure 3:**
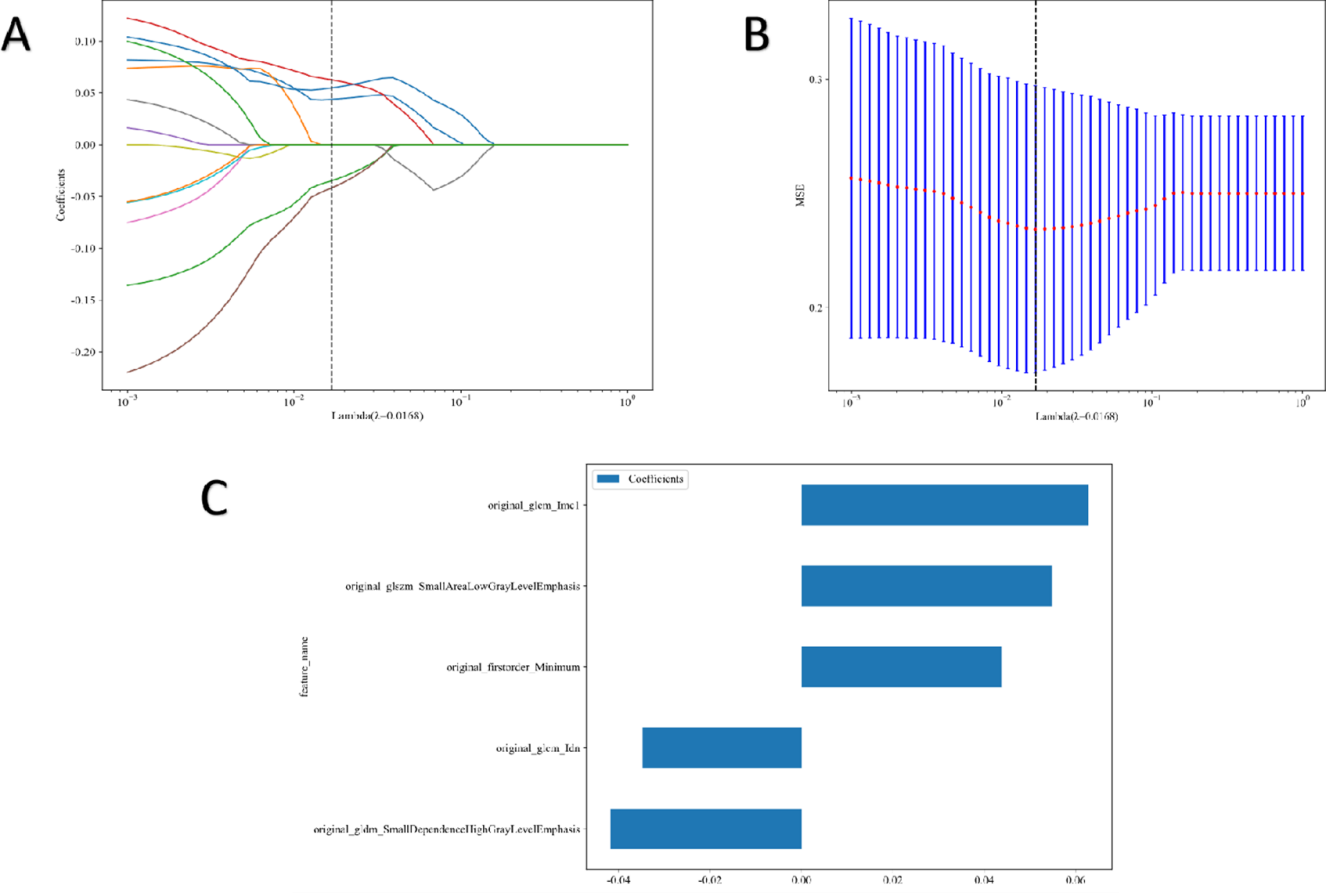
Radiomic feature selection. Coefficients of 10−fold cross validation **(A)** and mean standard error of 10−fold cross validation **(B)**. Final extracted radiomics features **(C)**.

**Figure 4:**
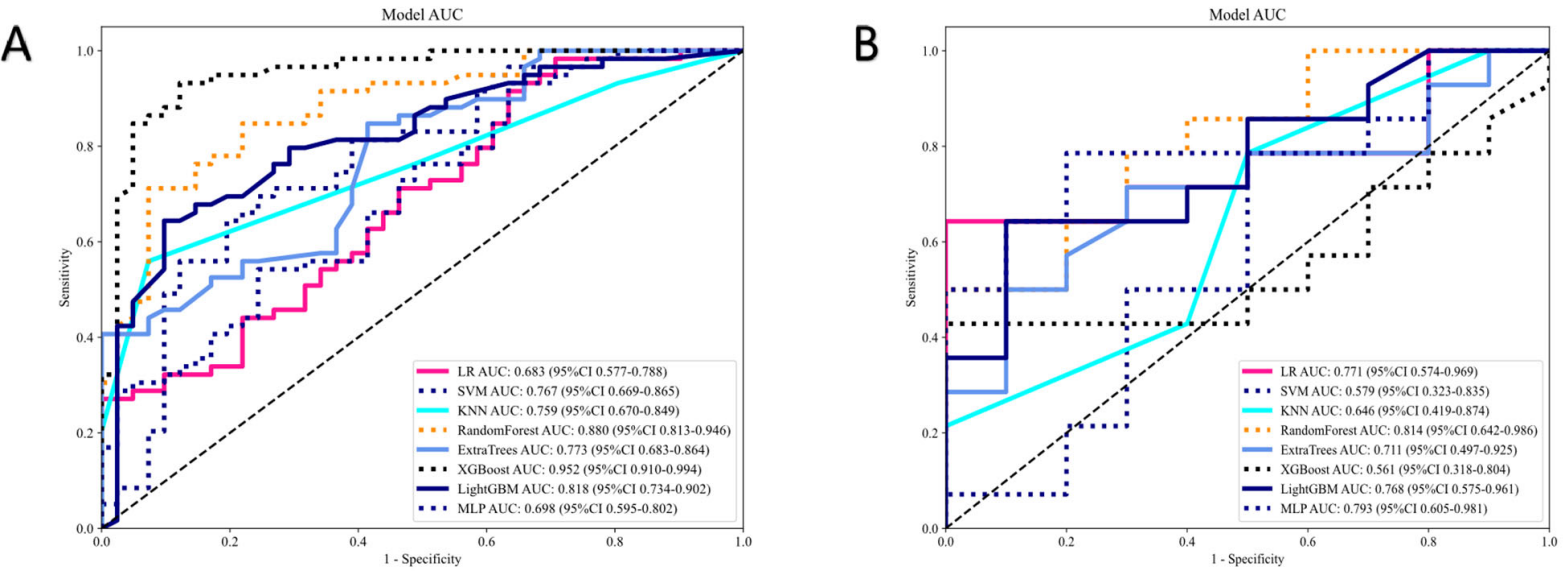
The effectiveness of radiomics models in the training cohort **(A)** and test cohort **(B)**.

Five features were selected to build the radiomics signature. Details of these features are illustrated in **Figure 3**. Eight models were built and compared to find the best-performing model. The results for all algorithms in the training and test sets are shown in **Table 3**; **Figure 4**. In both the training and test groups, the RF algorithm demonstrated the highest AUC values for predicting HER2 status in UBC, with AUCs of 0.880 (95% CI 0.813–0.946) and 0.814 (95% CI 0.642–0.986), respectively.

**Table 3:** Characteristics of all radiomics models.

Model	Cohort	AUC	Accuracy	Sensitivity	Specificity
LR	Training cohort Test cohort	0.683 (0.577 - 0.788) 0.771 (0.574 - 0.969)	0.680 0.750	0.898 0.571	0.366 1.000
SVM	Training cohort Test cohort	0.767 (0.669 - 0.865) 0.579 (0.323 - 0.835)	0.710 0.625	0.678 0.714	0.756 0.500
RF	Training cohort Test cohort	0.880 (0.813 - 0.946) 0.814 (0.642 - 0.986)	0.790 0.707	0.695 0.429	0.927 1.000
KNN	Training cohort Test cohort	0.759 (0.670 - 0.849) 0.646 (0.419 - 0.874)	0.530 0.500	0.203 0.429	1.000 0.600
ExtraTrees	Training cohort Test cohort	0.773 (0.683 - 0.864) 0.711 (0.497 - 0.925)	0.730 0.667	0.831 0.643	0.585 0.700
XGBoost	Training cohort Test cohort	0.952 (0.911 - 0.994) 0.561 (0.318 - 0.804)	0.900 0.625	0.915 0.357	0.878 1.000
LightGBM	Training cohort Test cohort	0.818 (0.734 - 0.902) 0.768 (0.575 - 0.961)	0.720 0.625	0.593 0.429	0.902 0.900
MLP	Training cohort Test cohort	0.698 (0.595 - 0.802) 0.793 (0.605 - 0.981)	0.620 0.750	0.525 0.714	0.756 0.800

AUC, area under the curve; LR, logistic regression; SVM, support vector machine; RF, random forest; KNN, k-nearest neighbors; ExtraTrees, extremely randomized trees; XGBoost, extreme gradient boosting; LightGBM, light gradient boosting machine; MLP, multi-layer perceptron.

### 3.3 Construction of the radiomics nomogram

**Figure 5:**
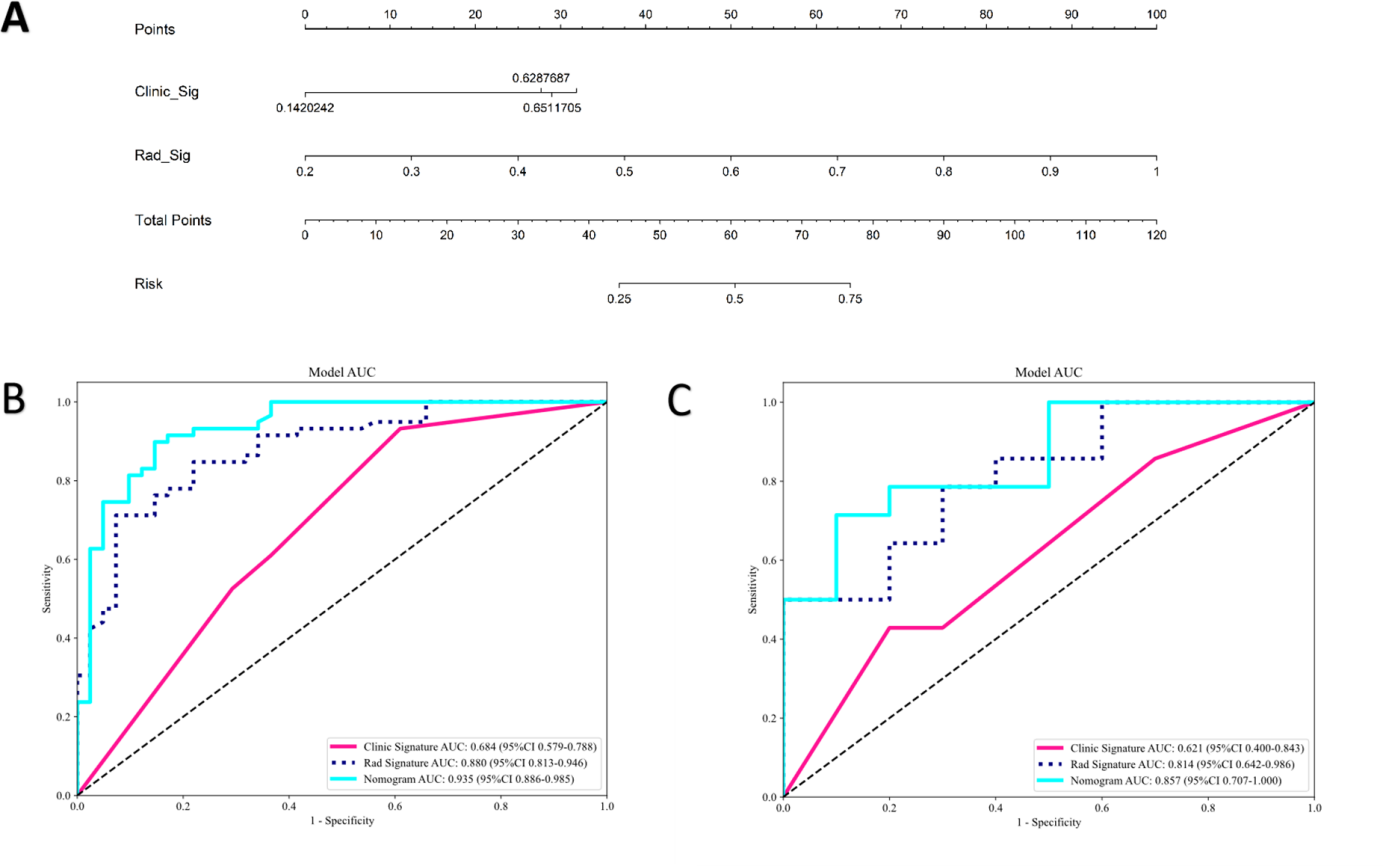
The radiomics-clinical nomogram **(A)** and ROC of clinical model, radiomics model and clinical radiomics nomogram in both training cohort **(B)** and test cohort **(C)**.

The clinical model was built using features with p<0.05 in our cohort. Only baseline gender and stalk met this criterion; therefore, these two features were employed to construct the clinical signature. The clinical model showed an AUC of 0.684 (95% CI 0.579–0.788) in the training group and 0.621 (95% CI 0.400–0.843) in the test group (**Table 4**; **Figure 5**).

**Table 4:** The effectiveness of clinical model, radiomics model and nomogram model.

Model	Cohort	AUC (95% CI)	Accuracy	Sensitivity	Specificity
Clinical	Training cohort Test cohort	0.684 (0.579 - 0.788) 0.621 (0.400 - 0.843)	0.620 0.417	0.610 0.000	0.634 1.000
Radiomics	Training cohort Test cohort	0.880 (0.813 - 0.946) 0.814 (0.642 - 0.986)	0.790 0.701	0.695 0.429	0.927 1.000
Nomogram	Training cohort Test cohort	0.935 (0.886- 0.985) 0.857 (0.707 - 1.000)	0.870 0.760	0.881 0.643	0.854 0.900

In the training group, the clinical-radiomics model exhibited an AUC of 0.935 (95% CI 0.886–0.985), with accuracy, sensitivity, and specificity values of 0.870, 0.881, and 0.854, respectively. In the test group, the AUC was 0.857 (95% CI 0.707–1.000), with corresponding accuracy, sensitivity, and specificity values of 0.760, 0.643, and 0.900, respectively (**Table 4**, **Figure 5**). The clinical-radiomics model demonstrated strong predictive performance for HER2 status, leading to the construction of a radiomics nomogram (**Figure 5**).

**Figure 6:**
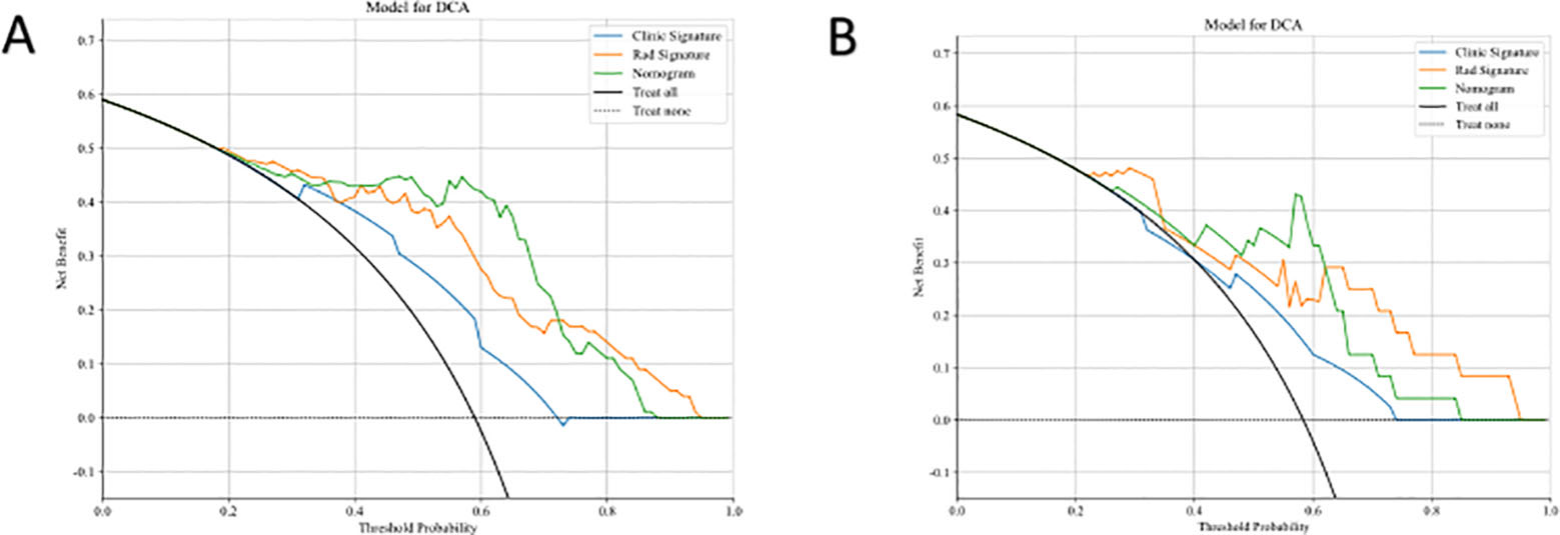
Comparison of the Efficiency of Three Models. DCA curves of three models in both training cohort **(A)** and test cohort **(B)**.

**Figure 6** displays the DCA results for the clinical, radiomics, and clinical-radiomics models. DCA indicated that the radiomics-clinical model could offer greater benefit in many scenarios.

Differences between the AUCs of the clinical-radiomics and clinical models were statistically significant in both the training and test groups (DeLong test; p<0.01). A significant difference was also found between the AUCs of the clinical-radiomics and radiomics models in the training group (DeLong test; p=0.022). However, in the test group, no significant difference was observed between the clinical-radiomics and radiomics models (DeLong test; p=0.095).

## 4 Discussions

In this study, we developed three predictive models (clinical model, radiomics model, and clinical-radiomics model) to predict HER2 status in patients with UBC. The results showed that the clinical model had the poorest performance (AUC=0.621 in the test set), followed by the radiomics model (AUC=0.814 in the test set). The combined model had the best performance (AUC=0.857 in the test set). Therefore, a radiomics nomogram based on CE-CT could be an effective and noninvasive tool for predicting the HER2 status in patients with UBC. The radiomics nomogram showed that HER2 status was associated with gender and tumor stalk. However, there is no literature to explore the relationship between tumor stalk and HER2 status in bladder cancer. About three-quarters of UBCs are papillary, and approximately sixty-six percent of these exhibit stalks (20). The pathological basis of the tumor stalk is composed of loose connective tissue with fibrotic tissue, capillaries, some inflammatory cells, and tissue edema (21). Kang et al. reported that the tumor stalk refers to hypo- or hyperattenuating structures on CE-CT during the corticomedullary to nephrographic phase (18). The typical appearance of tumor stalk on MRI was low-signal intensity on T2-weighted and diffusion-weighted images, and continuous submucosal enhancement at the base of the tumor on fat-suppressed dynamic contrast-enhanced MRI (22, 23). Several studies have found that bladder tumors with stalks are an important imaging feature in differentiating NMIBC (T1-stage or lower) from MIBC (T2-stage or higher) (23, 24). Bladder tumors without stalks tend to be of advanced T-stage (T2-stage or higher). A meta-analysis indicated that HER2 overexpression was significantly correlated with high tumor stage (25). Therefore, tumors without stalks may be correlated with HER2 overexpression. Additionally, integrating the radscore with tumor stalk could enhance predictive performance, providing an effective and noninvasive tool for clinical decision-making (23, 26). Tumors with stalks can be resected more easily and typically indicate a lower T-stage, which correlates with a better prognosis (22).

Accurately predicting HER2 status in patients with UBC is crucial because HER2-positive and HER2-negative cases have different clinical treatments and prognoses. In recent years, there has been significant progress in medical therapy for UBC. Following chemotherapy and immune checkpoint immunotherapy, anti-HER2 targeted treatments, such as monoclonal antibodies, small molecule tyrosine kinase inhibitors, and antibody-drug conjugates, have shown significant efficacy in locally advanced or metastatic HER2-positive urothelial carcinoma (27, 28). Additionally, anti-HER2 targeted therapy could be a viable option for high-risk and HER2-positive patients with NMIBC who have the highest risk of disease recurrence and progression after BCG therapy (11). Medical imaging is a common approach for preoperative cancer diagnosis; however, its accuracy in assessing HER2 status remains unsatisfactory. HER2 status is typically determined using invasive procedures like IHC or FISH, which can pose risks of infection, bleeding, and other complications. Moreover, IHC and FISH are time-consuming, labor-intensive, and expensive (29). Therefore, developing a noninvasive and effective method for accurately assessing HER2 status in patients with UBC is critical.

Currently, radiomics is at the forefront of medical imaging analysis, utilizing advanced techniques to extract quantitative features from radiographic images such as CT, MRI, or ultrasound scans. These radiomics features (such as first-order features, shape features, and textural features) contain a wealth of information beyond what is visible to the naked eye (30). Once extracted, these features are analyzed using statistical methods to build predictive models. Radiomics has shown promise in various medical applications, particularly in oncology. In cancer diagnosis and treatment, radiomics can help identify biomarkers associated with tumor aggressiveness, predict treatment response, assess patient prognosis, and more. By leveraging radiomics, clinicians can potentially tailor treatment plans to individual patients, leading to more effective and personalized care. Overall, radiomics represents a powerful tool for unlocking hidden information within medical images, enabling more precise diagnosis, treatment planning, and patient management across various medical specialties. Numerous reports have highlighted the utility of radiomics for the noninvasive prediction of various factors in BC, including pathological grade, staging, lymph node metastasis, Ki67 status, prognosis, and recurrence (16, 17, 31–33). Li et al. reported that the radiomics model achieved an AUC of 0.910 in the test set, demonstrating its potential usefulness for noninvasively and preoperatively assessing tumor grade in NMIBC (31). Feng et al. demonstrated that their clinical-radiomics model exhibited significant accuracy for the prediction of Ki67 status of bladder cancer in both the training and validation cohorts, achieving AUC values of 0.836 and 0.887, respectively (17). Park et al. used contrast-enhanced CT to construct a radiomics model to evaluate objective response and disease control of patients with metastatic urothelial carcinoma treated with immunotherapy targeting programmed cell death 1 (PD-1) and its ligand (PD-L1). The AUCs for objective response and disease control in the training cohorts were 0.87 and 0.77, whereas those in the validation cohorts were 0.87 and 0.88, respectively (16). To the best of our knowledge, there is only one report focused on radiomics features for predicting HER2 status in UBC. Yu et al. reported an MRI-based radiomics approach that can be used as a noninvasive tool to assess HER2 status in patients with UBC, with an AUC of 0.929 in the training cohort, 0.859 in the validation cohort, and 0.712 in the test cohort (15). However, no prior research has focused on CT radiomics for predicting HER2 status in patients with UBC.

In this study, we utilized eight machine learning-based classifiers (LR, SVM, RF, KNN, ExtraTrees, XGBoost, LightGBM, and MLP) for model construction. RF emerged as the top performer among these models in predicting HER2 status, with an AUC of 0.880 in the training group and 0.814 in the test group. RF is favored for its robustness, scalability, and ability to handle complex datasets and diverse tasks effectively. Its flexibility and ability to provide insights into feature importance make it a valuable tool in machine learning applications. Based on prior research, RF has demonstrated remarkable performance compared with other machine learning algorithms in various prediction tasks, such as predicting preeclampsia, in-hospital mortality for critically ill patients with sepsis-associated acute kidney injury, telomerase reverse transcriptase (TERT) promoter mutation status in adult glioblastoma, and meningioma grade (34–37). Therefore, we believe RF to be a dependable classifier in radiomics, offering broad applicability for future research endeavors. We established a nomogram model based on radiomics and clinical features for the noninvasive prediction of HER2 status in patients with UBC. Compared with the clinical model and the radiomics signature, the nomogram model enhanced the prediction of HER2 status. In our study, the nomogram model (AUC=0.935, 0.857) performed better than the radiomics model (AUC=0.880, 0.814) and the clinical model (AUC=0.684, 0.621) in both the training and test cohorts. The results indicate that the nomogram can be used to noninvasively assess HER2 status in UBC, which may help clinicians in more precisely discriminating between HER2-positive and HER2-negative patients with UBC.

A radiomics nomogram is a graphical tool used to predict the probability or outcome of specific events. They integrate multiple predictive factors and quantify risk by mapping them onto an easily understandable score or scale. Nomograms are commonly used in medical and statistical fields to assist physicians and researchers in making personalized predictions and decisions, such as forecasting patient survival rates or the risk of disease progression. However, nomograms have several limitations in their prediction capability. First, there are currently no universal reporting standards for nomograms (38). Different interpretations or judgments by different constructors can lead to variations in the nomogram’s accuracy and usability. Second, nomograms are suited for linear or simple nonlinear relationships between variables. If the relationship becomes highly nonlinear or involves complex functions, a nomogram may not be practical or accurate. Third, while radiomic nomograms provide predictive models, interpreting the contributions of individual radiomic features to the predictions can be challenging. Many radiomic features may lack direct clinical interpretation, making it difficult for clinicians to understand the biological or pathological significance behind the predictions made by the nomogram. Finally, traditional nomograms are static and cannot easily be updated (39). In technology-rich environments, electronic risk assessment tools can be developed for dynamic predictions, effectively mitigating many limitations associated with traditional nomograms (40).

However, there are several limitations to this study. Firstly, the relatively small sample size constrains the actual performance of the models. Secondly, it is conducted at a single center and lacks an external dataset for validation purposes. Thirdly, the study design is retrospective, which may lead to selection bias. Despite these limitations, integrating conventional clinical factors with radiomic signatures could potentially enhance the diagnostic prediction of HER2 expression status. The next phase of our work will focus on developing prospective, multicenter studies with larger sample sizes.

## 5 Conclusions

In summary, the nomogram integrating clinical features and radiomics signatures demonstrated outstanding diagnostic efficacy in distinguishing HER2-positive and HER2-negative UBC. The nomogram model displayed higher AUC and accuracy levels than the radiomics and clinical models, offering a promising tool for urologists and oncologists to formulate more effective personalized treatment strategies.

## Data Availability

The raw data supporting the conclusions of this article will be made available by the authors, without undue reservation.
